# Effects of Sulfur Dioxide on Disease Resistance and Storage Quality of Durian (*Durio zibethinus* L.): A Comprehensive Analysis Integrating GC-IMS, HTS, Physiological and Biochemical Parameters

**DOI:** 10.3390/foods15193574

**Published:** 2026-10-08

**Authors:** Shuangdi Hou, Xia Wang, Hui Liu, Phebe Ding, Xian Ji, Xiaoshuan Zhang, Zhiqiang Zhu

**Affiliations:** 1 Key Laboratory of Postharvest Physiology and Storage of Agricultural Products, Institute of Agricultural Products Preservation and Processing Science and Technology, National Engineering Technology Research Center for Preservation of Agricultural Product, Tianjin Academy of Agricultural Sciences, Ministry of Agriculture and Rural Affairs, Tianjin 300384, China; 2 College of Food Science and Nutritional Engineering, China Agricultural University, Beijing 100091, China; 3 China Packing Research & Test Center, Tianjin 300457, China; 4 Department of Crop Science, Faculty of Agriculture, Universiti Putra Malaysia (UPM), Serdang 43400, Selangor, Malaysia; phebe@upm.edu.my; 5 Sanya Research Institute of China Agricultural University, Sanya 572000, China

**Keywords:** sulfur dioxide, durian, flavor, microbial communities, disease resistance

## Abstract

In this study, we examined the multifaceted effects of sulfur dioxide (SO_2_) on the postharvest preservation of durian and the underlying potential mechanism of disease resistance. Our results show that 2% SO_2_ preserved the good quality of durian by inhibiting its respiration rate and ethylene production (day 15), reducing weight loss, and maintaining color, firmness, and ascorbic acid content. Furthermore, the volatile organic compounds (VOCs) were investigated further by gas chromatography-ion mobility spectrometry (GC-IMS). Ester compounds (fruity aroma of durian) were primarily intensified in SO_2_-treated samples, while SO_2_ weakened the content of volatile sulfur compounds. Certain antioxidant and antifungal VOCs, including alpha-terpinene, benzyl alcohol, 2-decanone, and 2-heptylfuran, may contribute to enhancing the disease resistance of durian. High-throughput sequencing (HTS) analysis indicated that SO_2_ may enhance the disease resistance of durian by shifting the fungal community succession, which stabilized the relative abundance of *Meyerozyma* sp. and decreased the relative abundance of *Alternaria* sp. (on day 5), *Verticillium* sp. (on day 5), and *Moesziomyces* sp. (on day 5 and 10). Additionally, SO_2_ induced the activities of defense-related enzymes, such as catalase (CAT), polyphenol oxidase (PPO), peroxidase (POD), chitinase (CHI), and β-1,3-glucanase (β-GLU), which may synergize with antifungal VOCs and fungal community succession to enhance durian disease resistance. These results clarify the potential distinct mechanisms of disease resistance of SO_2_ and provide a scientific basis for the application of SO_2_ in the postharvest preservation of durian fruits.

## 1. Introduction

Durian (*Durio zibethinus*), widely acknowledged as the king of tropical climacteric fruits in Southeast Asia, is characterized by a spiny husk and a unique flavor, whose odor is often described as a sulfurous, onion-like aroma [1]. However, the shelf life of durian is critically short due to its high respiratory rate and rapid ethylene production, which accelerate over-ripening, husk cracking, microbial infection, and flavor deterioration [2]. This severe limitation presents a major obstacle to long-distance marketing and the industrial development of this fruit. Therefore, developing effective strategies to retard senescence and safeguard the commercial attributes of durian fruit, while elucidating the underlying preservation mechanism, constitutes a major challenge in contemporary postharvest research.

SO_2_ is a chemical additive extensively employed in the food industry. Despite ongoing safety controversies, SO_2_ remains indispensable in the fruit and vegetable industry [3]. The most distinctive feature of durian is its unique volatile flavor compounds, primarily sulfur-containing compounds and esters [4]. Studies have shown that appropriate concentrations of SO_2_ can activate the ethanol metabolic pathway in grapes, thereby effectively enhancing the contents of ester aroma compounds. Concurrently, SO_2_ upregulates the expression of flavor-related genes, thereby promoting the biosynthesis of flavor metabolites [5]. However, inappropriate SO_2_ application is a primary factor responsible for flavor deterioration in fruits. The reaction of SO_2_ with endogenous fruit components yields sulfur-containing compounds with offensive odors. For instance, SO_2_ treatment results in a marked elevation of glucosinolates and related sulfur-containing metabolites, which may obscure the original fruity and floral aromas [6]. Notably, some VOCs not only influence fruit flavor but also possess antibacterial properties, such as 2-decanone [7] and benzyl alcohol. Although studies have shown that combined treatment with SO_2_ and CO_2_ can maintain durian firmness, delay weight loss, reduce rot rate, and strengthen disease resistance by improving the activities of POD and CAT [8], the effects of SO_2_ treatment on durian flavor remain unclear.

The efficacy of SO_2_ in preserving fruits is largely attributable to its broad-spectrum antimicrobial properties and antioxidant capacity [9]. Specifically, it serves to inhibit and destroy a range of surface-borne microorganisms, such as spoilage molds, yeasts, and pathogenic bacteria, thereby extending the shelf life of fruits [10]. For example, it was reported to reduce the survival of *Escherichia coli*, *Salmonella* spp., and *Listeria moncytogenes* on dried apricots [11]. Similarly, SO_2_ could kill *Botrytis cinerea* conidia and inactivate mycelia of grapes [12]. In addition, some other mold species such as *Cladosporium* sp. *Penicillium* sp., and *Alternaria* sp. were sensitive to SO_2_ and its derivates antimicrobial activity [13]. Except for the direct interaction with pathogens, SO_2_ can modulate the antioxidant defense system, cell wall modification and the synthesis of stress proteins in plant tissue [14]. Previous studies have demonstrated that SO_2_ can activate the activities of defense enzymes (phenylalanine ammonia-lyase (PAL), PPO, CHI, and β-GLU), with accompanying up-regulation of pathogen-related protein genes (*CHI* and *GLU*) expression, thereby enhancing the disease resistance of fruits [15]. Extensive research has confirmed that altering the microbial diversity on the fruit surface can effectively reduce the risk of postharvest spoilage and contamination by pathogenic fungi [16,17]. At present, investigations into the mechanism of direct action of SO_2_ focus on “surface sterilization”. However, there are few reports on how it regulates the structure of the microbial community on the surface of durians. To explore the potential coordinated responses between microbial community succession and enzymatic defense responses in SO_2_-treated fruit could provide a scientific basis for its application in enhancing the disease resistance of durian.

In this study, the effects of SO_2_ on respiration rate, ethylene production rate, weight loss, firmness, color, total soluble solids (TSS) and ascorbic acid content were analyzed in durian during storage at 14 °C. Additionally, durian flavor was analyzed using GC-IMS, while HTS technology was used to reveal the effect of SO_2_ on the diversity and community succession of the husk microbiome. The main aim is to comprehensively analyze the overall effects of SO_2_ treatment on fungal community composition and dynamics, and to understand the relationship among fungal communities, VOCs, and defense-related enzymes. This study provides new insights into the potential mechanisms by which SO_2_ treatment improves the postharvest quality of durian.

## 2. Material and Methods

### 2.1. Fruit and SO_2_ (TS) Treatment

Durian fruits (variety: ‘Monthong’) with commercial maturity (yellow pulp, average weight of 2.5 ± 0.5 kg) were procured from the Shouheng Gaobeidian International Agricultural Products Trading Center in Baoding City, Hebei Province, China. Following inspection, fruits were confirmed to be devoid of diseases, pests and physical damage. Subsequently, they were transported to the Tianjin Academy of Agricultural Sciences (China) within two hours and divided randomly into two groups. The first group served as the control without any treatment, while the second group underwent SO_2_ (TS) treatment. Every three durians were placed in a sealed box (503 × 323 × 298 mm, 48.42 L), into which 0.97 L of SO_2_ gas was injected to achieve a 2% concentration. The SO_2_ gas was supplied by Tianjin Jinxi Huanda Chemical Gas Operation Department, China. Fumigation was conducted in a ventilated area with appropriate personal protective equipment. After a 30 min fumigation process, the durians were left exposed at 26–28 °C (ventilated). These durians were grouped and stored at 14 °C and 80% relative humidity (RH). The storage room was equipped with an internal air-circulation system. A total of 42 fruits were used. Six fruits were used as the initial samples (control sample, untreated with SO_2_) on day 0. On days 5, 10 and 15, six fruits were randomly chosen from each group for analysis of relevant indicators. Then, they were frozen and stored in liquid nitrogen. The frozen tissue samples were stored at −80 °C for subsequent assessment of pertinent enzyme activities.

### 2.2. Total Fungi Counts and SO_2_ Residue

The total number of fungi was determined using Potato Dextrose Agar (PDA) (Shanghai Bowei Microbiology Technology Co., Ltd.: Shanghai, China) according to the method described in GB 4789.15-2016 (National Food Safety Standard: Food Microbiological Examination-Enumeration of Molds and Yeasts) [18]. A 25 g sample was homogenized with 225 mL of sterile diluent for 1–2 min to prepare a 1:10 homogenate, followed by serial 10-fold dilutions. For each dilution, 1 mL of the homogenate was plated in triplicate. Plates were incubated at 28 ± 1 °C for 5 days under aerobic conditions, and colonies were observed. SO_2_ residue was assayed following the instructions of SO_2_ rapid detection kits (Shanghai Zerui Chemical Technology Co., Ltd., Shanghai, China). 1.0 g durian pulp was homogenized with 10 mL distilled water. Let it stand for 15 min, then centrifuge at 8000 rpm for 15 min. Transfer 1 mL of the supernatant to a test tube, add 2 drops each of test solutions A, B, and C in sequence, mix well, let it stand for 5 min, and compare the color with the color chart.

### 2.3. Stored Durian Quality Analysis

#### 2.3.1. Respiration Rate and Ethylene Production Rate

The respiration rate and ethylene production rate of durian pulp were measured according to [19]. A 500 g sample was placed into a 3.0 L sealed polypropylene (PP) box, the lid of which was fitted with a rubber septum to facilitate syringe injection (Shanghai Bolige Industry and Trade Co., Ltd., Shanghai, China). After equilibration at 25 °C for 2 h, 1 mL of headspace gas was withdrawn using a sampler and injected into a gas chromatograph (GC-2010 Plus, Shimadzu, Kyoto, Japan) for ethylene determination. Simultaneously, CO_2_ consumption was monitored with a gas composition analyzer (CA-10, Sable System International, North Las Vegas, NV, USA), and the respiration rate was calculated accordingly.

The respiration rate was calculated as

$$\mathrm{Respirationrate}\left(\mathrm{mg}\cdot {\mathrm{kg}}^{-1}\cdot h^{-1}\right)=\frac{\varphi \times V\times 44\times 1000}{100\times m\times t\times 24.5}$$

where φ is the CO_2_ volume fraction (%), V is the headspace volume (L), m is the sample mass (kg), t is the sealing time (h), and 24.5 is the molar volume of gas at 25 °C (L mol^−1^).

The ethylene production rate was calculated as

$$\mathrm{Ethyleneproductionrate}\left(\mu L\cdot {\mathrm{kg}}^{-1}\cdot h^{-1}\right)=\frac{X\times B_{2}\times V}{B_{1}\times m\times t}$$

where X is the ethylene standard volume fraction (‰), B_1_ is the standard peak area, B_2_ is the sample peak area, V is the container volume (L), m is the sample mass (kg), and t is the sealing time (h).

#### 2.3.2. Weight Loss Rate, Color and Firmness

Weight loss rate of durian was defined as the ratio of weight change to initial weight, measured at 0, 5 and 10 days. Color of durian pulp was measured via a calibrated precision colorimeter (CR-400, Konica Minolta Sensing, Sakai, Osaka, Japan; calibrated with a black-and-white plate), yielding L* (lightness), a* (redness-greenness) and b* (yellowness-blueness) values. Firmness was assessed by TPA on a texture analyzer (TAXTPLUS, Stable Micro Systems, Godalming, Surrey, UK) using a 75 mm probe, under a compression and trigger force of 0.049 N and speeds of 5 mm·s^−1^ (pre-test), 2 mm·s^−1^ (test) and 2 mm·s^−1^ (post-test), with the final expressed as N·cm^−2^.

#### 2.3.3. TSS and Ascorbic Acid

TSS and ascorbic acid content of durian pulp were determined according to [19], with slight modifications. Durian pulp was homogenized with deionized water at a ratio of 1:3, and then centrifuged at 4 °C and 12,000 rpm for 25 min. The supernatant was collected, and TSS content was measured using a sugar refractometer (PAL-BXIACID F5, ATAGO, Tokyo, Japan). TSS was calculated as

TSS(%) = B × D

where B is the refractometer reading and D is the dilution factor.

For ascorbic acid analysis, the durian pulp was homogenized with 20 g L^−1^ oxalic acid solution at a ratio of 1:1, then filtered and titrated with sodium 2,6-dichlorophenolindophenol solution until a color change was observed. The volume of titrant used was recorded, and the ascorbic acid content was calculated accordingly. The ascorbic acid content was calculated as

$$\mathrm{Ascorbic}\;\mathrm{acid}(\mathrm{mg}/100\;g)=\frac{C\times V_{1}}{w\times V_{2}}$$

where C is the ascorbic acid content in the measured sample solution (mg), V_1_ is the total volume of the sample solution (mL), w is the sample mass (mg), and V_2_ is the volume of sample solution used for measurement (mL).

### 2.4. GC-IMS Analysis

VOCs in durian were analyzed using a FlavourSpec^®^ GC-IMS instrument (G.A.S. Gesellschaft für analytische Sensorsysteme mbH, Dortmund, Germany) equipped with an MXT-5 capillary column (15 m × 0.53 mm, RESTEK, Bellefonte, PA, USA), following the methods of [20], with minor modifications. A 3 g sample was placed in a 20 mL headspace vial and incubated at 40 °C for 10 min. Subsequently, 500 μL of headspace gas was injected at 60 °C. Nitrogen (99.999% purity) served as the carrier gas, with the flow program configured as follows: an initial flow rate of 2 mL/min maintained for 2 min, followed by an increase to 10 mL/min over 10 min, and a further escalation to 100 mL/min over 20 min. Volatile organic compounds were identified by comparing their retention indices and drift times with those in the GC-IMS library (NIST 2014).

### 2.5. Fungal Community Analysis

#### 2.5.1. Collection of Surface Fungal Communities

Every five days during storage at 14 °C, 300 g of durian husk was washed with 500 mL of sterile distilled water in a conical flask. The washings were collected and vacuum-filtered through a microporous membrane (50 mm diameter, 0.45 µm pore size) to obtain microorganisms. The microbes retained on the membrane were subsequently transferred to sterile centrifuge tubes with sterile forceps and stored at −80 °C for subsequent ITS1 amplicon sequencing.

#### 2.5.2. DNA Extraction, PCR Amplification, and ITS Sequencing

Genomic DNA was extracted from the samples using the method of CTAB [21]. Specific primers (ITS-1737 F: 5′-GGAAGTAAAAGTCGTAACAAGG-3′, ITS-2043 R: 5′-GCTGCGTTCTTCATCGATGC-3′) were employed to amplify distinct regions of ITS genes. All PCR reactions were conducted using Phusion^®^ high-fidelity PCR premixes (New England Biolabs). The 400–450 bp PCR products were mixed in equal proportions and purified using the Qiagen gel extraction Kit (Qiagen, Hilden, Germany). Follow the manufacturer’s recommendations and generate sequencing libraries using the TruSeq^®^DNA CR-Free sample Preparation Kit (Illumina, San Diego, CA, USA). The quality of the gene bank was evaluated on the Qubit@2.0 fluorometer (Thermo Fisher Scientific (Invitrogen), Carlsbad, CA, USA) and Agilent Bioanalyzer 2100 system. Finally, the gene bank was sequenced on the Illumina NovaSeq6000 platform and generated 250 bp paired-end reads. After sequencing, the paired-end reads were merged using FLASH (V1.2.11).

#### 2.5.3. ITS Bioinformatics Analysis

According to the QIIME (V1.7.0) quality control process, the original labels were subjected to quality filtering to obtain high-quality clean labels. The tags were compared with the Unite database “https://unite.ut.ee/ (accessed on 7 January 2026)”, and chimeric sequences were removed. Sequence analysis was conducted using Uparse software (Uparse v7.0.1001) [22]. Sequences with a similarity of ≥97% are assigned to the same OTU (Operational Taxonomic Unit). Based on the typical sequence annotation Unite database “https://unite.ut.ee/ (accessed on 7 January 2026)” through the Blast algorithm (QIIME software, 1.7.0) “http://qiime.org/scripts/assign_taxonomy.html (accessed on 7 January 2026)”. Meanwhile, multiple sequence alignments were performed using MUSCLE software (version 3.8.31) [23]. Finally, the data of each sample were homogenized, with the sample with the least data volume as the standard for homogenization. Beta diversity analysis was conducted using Qiime software (Version 1.9.1) based on weighted and unweighted distances. Based on the top 10 species in terms of abundance for each sample at different taxonomic levels (phylum, class, order, family, genus, species), a distribution histogram of relative abundance in Perl is plotted using SVG functions. Venn diagrams are generated in R using the Venn Diagram function and in Perl using the SVG function.

### 2.6. Defense-Related Enzyme Activities

All the enzyme activities (CAT, PAL, PPO, POD, CHI and β-GLU) were assayed following the instructions of enzyme assay kits (Solarbio Science Technology Co., Ltd., Beijing, China). Approximately 0.1 g of durian pulp was homogenized with 1 mL of extraction buffer in an ice bath, centrifuged at 8000–12,000 g for 10–20 min (CAT and POD: 8000× *g*, 10 min; PAL: 10,000× *g*, 10 min; PPO and β-GLU: 12,000× *g*, 10 min; CHI: 10,000× *g*, 20 min) at 4 °C, and the supernatant was collected for enzyme assays. All enzyme activities were measured in triplicate.

CAT (BC0205): Activity was measured by the decrease in absorbance at 240 nm due to H_2_O_2_ decomposition:

$$\mathrm{CAT}\;\mathrm{activity}\;(U\;g^{-1}\;\mathrm{fresh}\;\mathrm{weight})=\frac{764.5\times \Delta A}{W}\times F$$

where ΔA is the absorbance change, W is sample mass (g), and F is the dilution factor. One unit was defined as 1 μmol H_2_O_2_ degraded per minute per g tissue.

PAL (BC0215): Activity was measured by the increase in absorbance at 290 nm due to trans-cinnamic acid formation:

$$\mathrm{PAL}\;\mathrm{activity}\;(U\;g^{-1}\;\mathrm{fresh}\;\mathrm{weight})=\frac{26.67\times \Delta A}{W}$$

One unit was defined as 0.05 absorbance change per minute per g tissue.

PPO (BC0195): Activity was measured by the increase in absorbance at 410 nm due to catechol oxidation:

$$\mathrm{PPO}\;\mathrm{activity}\;(U\;g^{-1}\;\mathrm{fresh}\;\mathrm{weight})=\frac{120\times \Delta A}{W}\times F$$

One unit was defined as 0.005 absorbance change per minute per g tissue.

POD (BC0095): Activity was measured by the increase in absorbance at 470 nm due to guaiacol oxidation:

$$\mathrm{POD}\;\mathrm{activity}\;(U\;g^{-1}\;\mathrm{fresh}\;\mathrm{weight})=\frac{9800\times \Delta A}{W}\times F$$

One unit was defined as 0.005 absorbance change per minute per g tissue.

CHI (BC0825): Activity was measured at 585 nm based on N-acetylglucosamine release from chitin:

$$\mathrm{CHI}\;\mathrm{activity}\;(U\;g^{-1}\;\mathrm{fresh}\;\mathrm{weight})=\frac{2.5\times x}{W}$$

where x is the N-acetylglucosamine concentration (μg/mL) from the standard curve. One unit was defined as 1 μg N-acetylglucosamine produced per hour per g tissue.

β-GLU (BC0365): Activity was measured at 540 nm based on reducing sugar release from laminarin:

$$\beta \mathrm{-GLU activity}(U\;g^{-1}\;\mathrm{fresh}\;\mathrm{weight})=\frac{x}{W}\times F$$

where x is the reducing sugar concentration (mg/mL) from the standard curve. One unit was defined as 1 mg reducing sugar produced per hour per g tissue.

### 2.7. Statistical Analysis

All trials adopted a completely randomized design. All data were expressed as mean ± standard deviation (SD). The data were processed by single-factor analysis (ANOVA) with Duncan’s multiple range test using SPSS 27. An independent-samples *t*-test (two-tailed) was used at a significance level of *p* < 0.05.

## 3. Results and Discussion

### 3.1. Changes in Fruit Quality

#### 3.1.1. Screening for SO_2_ Concentration and Analysis Residue

**Figure 1:**
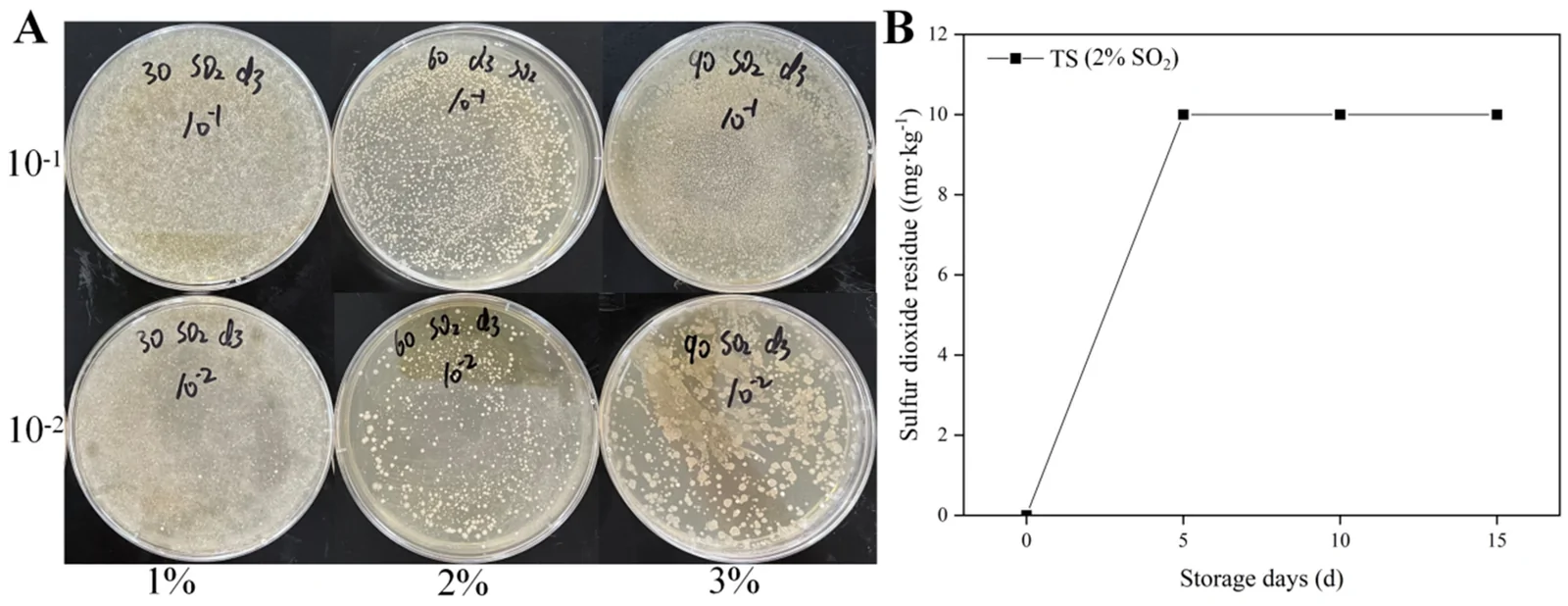
Screening for SO_2_ concentration and analysis of residue during storage. Antibacterial effect (**A**), fungal growth in culture plates after treating durian husk with different SO_2_ concentrations at the same dilution gradients (10^−1^ and 10^−2^); SO_2_ residue (**B**).

To determine the optimal SO_2_ fumigation concentration, a preliminary experiment was conducted at room temperature (30 °C). Durian husks were fumigated with 1%, 2%, and 3% SO_2_ and maintained under identical conditions until day 3. As illustrated in Figure 1A, the total fungal counts on the durian husk followed this order under the same dilution gradient: 1% > 3% > 2%. Notably, the 2% SO_2_ fumigation exhibited the most effective antifungal properties, resulting in the lowest mold and yeast count. The increased fungal count observed with the 3% SO_2_ treatment may be attributed to excessive SO_2_, which can damage the cell membrane, disrupt intracellular acidity, and accelerate plasmolysis. This damage to the cell membrane and wall may result in the release of intracellular substances, thereby creating a favorable environment for microbial growth and facilitating the extensive proliferation of pathogens [24]. The SO_2_ residue in the pulp remained below 10 mg·kg^−1^ after day 5 of storage under the 2% SO_2_ fumigation treatment (Figure 1B), which complies with the limit set by the Ministry of Agriculture of the People’s Republic of China [25]. Therefore, 2% SO_2_ fumigation (TS) was selected for the treatment of whole durian fruits.

#### 3.1.2. Effect of SO_2_ (TS) Treatment on Respiration Rate and Ethylene Production Rate

**Figure 2:**
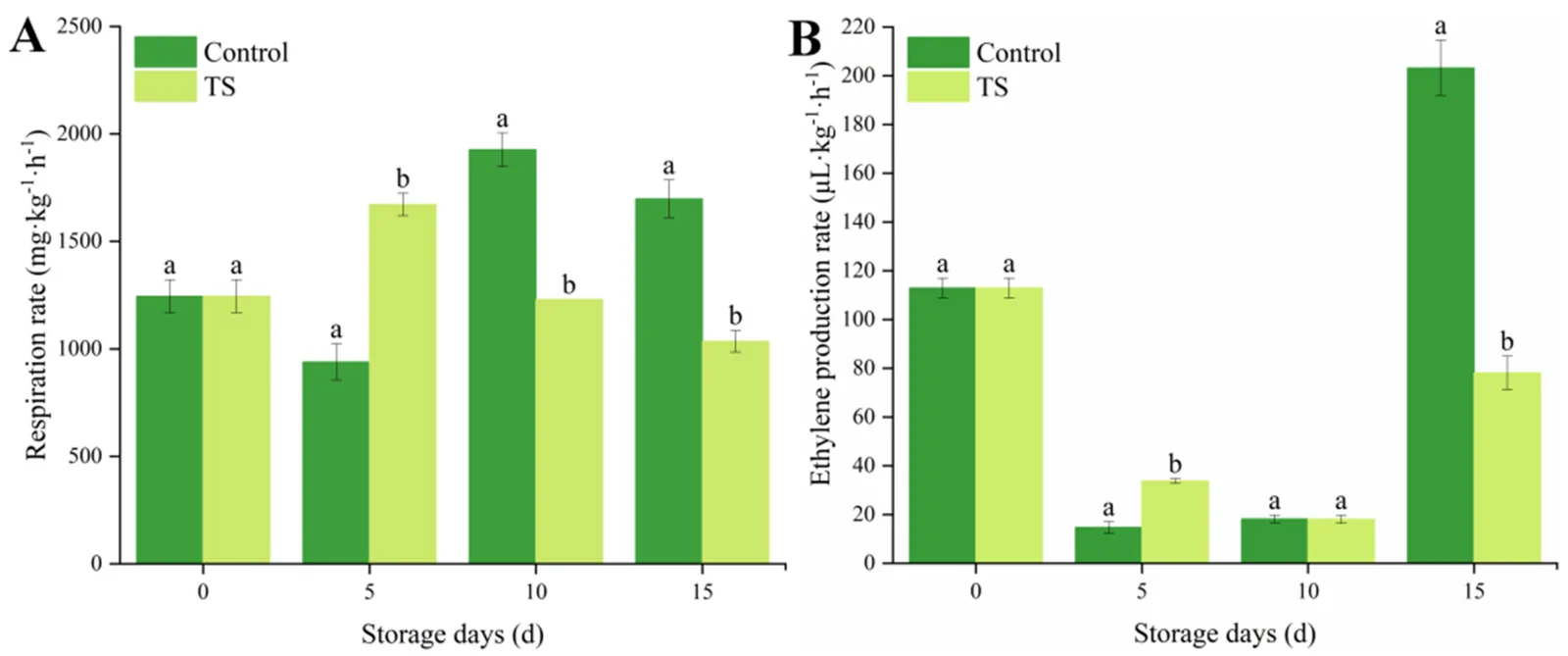
Effect of different treatments on respiration rate (**A**) and ethylene production rate (**B**). Data are means ± SD of three biological replicates. Within the same storage day, different letters indicate significant differences between the control and TS samples (*p* ˂ 0.05; all pairs compared using an independent-samples *t*-test (two-tailed)).

Respiratory metabolism plays a vital role in the preservation of fruit. As shown in Figure 2A, the respiration rate of durian pulp was 1246.73 mg·kg^−1^·h^−1^ at day 0 (room temperature, 30 °C). A decrease in the respiration rate of the control group was observed on day 5, which was ascribed to the temperature reduction from room temperature to 14 °C, followed by an increase on day 10 and a subsequent decrease on day 15. In the TS-treated fruit, the respiration rates increased initially and then decreased. Differing from the control group, the TS treatment did not exhibit a lower respiration rate on day 5 than on day 0; it reached a peak on day 5. This result is similar to [26]; they have shown that SO_2_ activates the tricarboxylic acid cycle and electron transport chain, thereby transiently promoting aerobic respiration, but the exact mechanism still needs further verification. After day 10, the respiration rates of the TS-treated sample were significantly lower than those in the control (*p* < 0.05), which consequently helps retard durian ripening and senescence. The results align with [3], suggesting that SO_2_ may function as an abiotic stressor that induces a compensatory respiratory burst in fruit tissues prior to the onset of metabolic suppression.

Ethylene, the primary hormone driving fruit ripening and senescence [27], exhibited a production rate of 112.95 μL·kg^−1^·h^−1^ in durian pulp on day 0, declining over the first 10 days before sharply rising by day 15 (Figure 2B). By day 5, the ethylene production rate in the TS group was significantly higher (*p* < 0.05) than in the control group, correlating with the respiratory rate (Figure 2A). On day 10, the control group showed a rapid increase in respiration rate, while low temperature stress continuously suppressed ethylene biosynthesis, preventing the climacteric peak. Similarly, ethylene production in TS-treated durian fruit at low temperature was also inhibited, mirroring the control group.

#### 3.1.3. Effect of SO_2_ (TS) Treatment on Weight Loss Rate, Firmness, Color, TSS and Ascorbic Acid

The weight loss rate serves as a crucial indicator of fruit storage quality, as it encompasses both transpirational water loss and respiratory dry matter consumption, thereby reflecting tissue water retention capacity and overall postharvest metabolic activity [28]. As shown in Table 1, the weight loss rate increased with storage duration across all samples, achieving maximum values of (15.83 ± 0.35) and (11.86 ± 1.16) in control and TS-treated fruit, respectively, by day 15. This increase is likely due to SO_2_ fumigation, which may influence stomatal aperture or modify the cuticular barrier properties of durian fruit, a mechanism akin to that observed in SO_2_-treated longan fruit [29]. Concurrently, the firmness of durian pulp diminished during storage, with TS-treated fruit exhibiting significantly higher firmness than control fruit prior to day 10 (*p* < 0.05). Both the L* and b* values decreased in control fruit over time, while these values in TS-treated fruit declined before day 10 and subsequently increased on day 15. Specifically, the L* and b* values in TS-treated fruit were significantly greater than those in control fruit after 10 days (*p* < 0.05), which indicate that a better color maintain in durian pulp [30]. However, a* values in two groups were no significant differences. These findings suggest that SO_2_ treatment effectively reduces weight loss in durian, preserves color brightness, and delays the softening of durian pulp.

TSS serve as a primary indicator of fruit ripeness and nutritional quality [31]. Initially, TSS was 30.40%. In the control group, TSS saw a slight increase to 31.33% by day 5 due to sugar accumulation as durian reached advanced maturity [32], followed by a gradual decline to 23.20% by day 15. However, in the TS-treated group, TSS dropped to 24.00% by day 10 (a significant decrease compared to the control, *p* < 0.05), then surged to 30.13% by day 15, significantly surpassing the control (*p* < 0.05). These findings suggest that SO_2_ facilitated a balance between sugar utilization and nutritional flavor, thereby postponing durian fruit ripening by 10 days and preserving superior flavor during later storage stages. This contrasts with prior research indicating that 2.2% SO_2_ fumigation did not impact firmness and TSS in blueberries [33]. We hypothesize that, unlike the direct application of SO_2_ fumigation on delicate-skinned berries, the thick husk of durian enables a gradual release of the fumigant into the inner pulp, ensuring a sustained effect that progressively regulates enzymatic processes to influence durian quality.

Ascorbic acid effectively scavenges oxygen-free radicals, thereby aiding in the preservation and shelf-life extension of fruits and vegetables [34]. Initially, the durian pulp displayed the highest ascorbic acid content of (15.09 ± 0.41) mg·100 g^−1^ on day 0, which significantly decreased across all samples over time. TS-treated fruit exhibited a higher ascorbic acid content level and experienced a slower rate of decline compared to the control group. A similar protective influence of SO_2_ on ascorbic acid content has been documented in grapes, where SO_2_ effectively maintained ascorbic acid levels during storage, consequently enhancing the fruits’ antioxidant capacity [35]. These findings suggest that SO_2_ can retard the degradation of ascorbic acid, thereby improving the antioxidant capacity and preserving the freshness of durian pulp.

### 3.2. Effect of SO_2_ (TS) Treatment on Fruit VOCs

**Figure 3:**
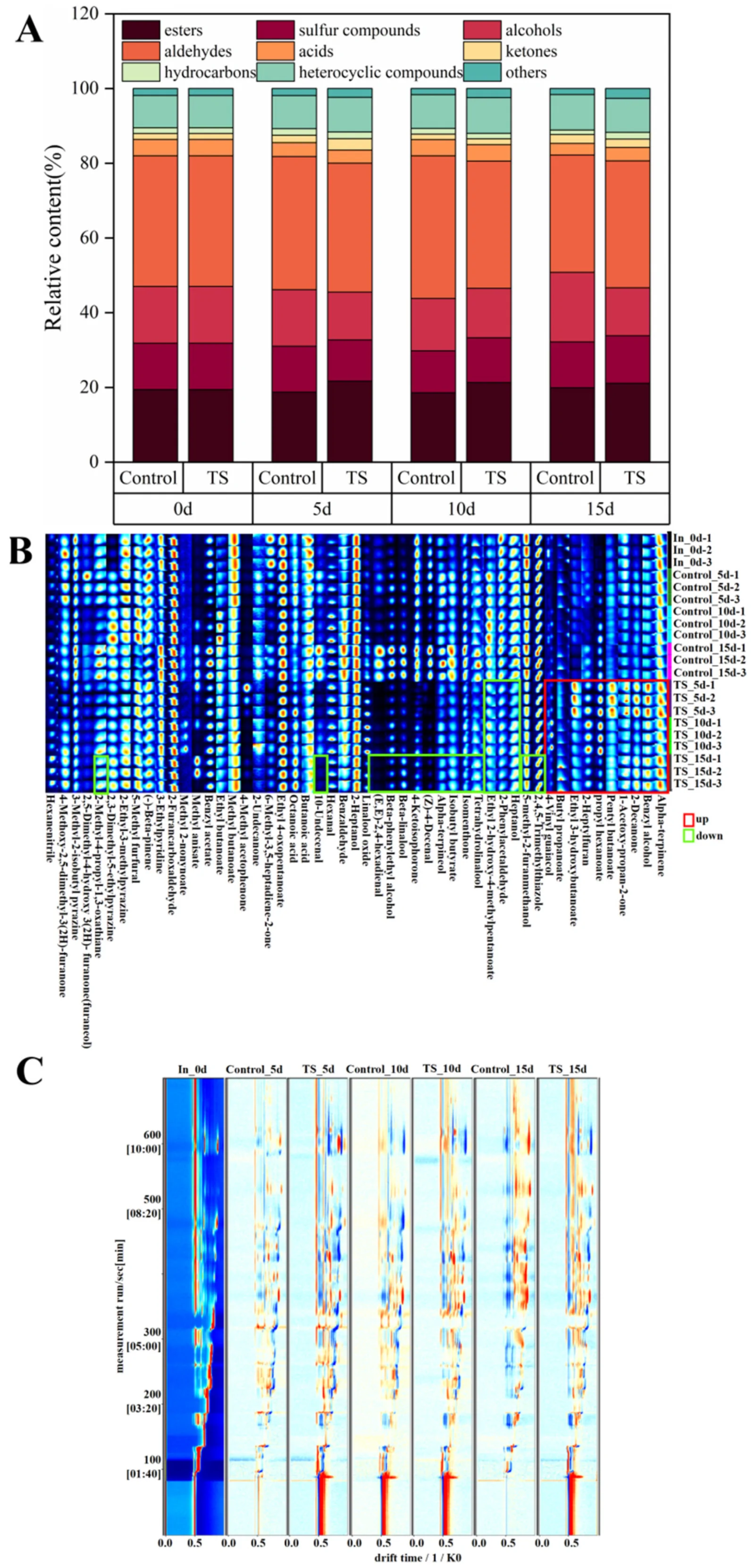
GC-IMS results of VOCs in durian. The relative content (%) of VOC classes (**A**), fingerprint gallery plot (**B**), and spectral difference plots (**C**).

VOCs are critical factors influencing both the unique flavor and postharvest quality of durian. To assess the impact of SO_2_ treatment on these flavor compounds, we utilized GC-IMS to profile the VOCs in the samples. A total of 51 VOCs were identified in ‘Montong’ durian fruit, with esters, sulfur compounds, alcohols, and aldehydes representing the primary volatile constituents (Figure 3A, Supplementary Materials, Table S1). These components establish the essential flavor characteristics of the samples.

The VOC fingerprint plot in Figure 3B, generated using the Gallery plugin, organizes samples in rows and VOCs in columns, employing a color scale from blue to red to indicate concentrations from low to high. Within the red region, compounds such as alpha-terpinene, benzyl alcohol, 2-decanone, 1-acetoxy-propan-2-one, pentyl butanoate, propyl hexanoate, 2-heptylfuran, ethyl 3-hydroxybutanoate, butyl propanoate and 4-vinyl guaiacol exhibited heightened signals in the T1 group. Specifically, esters (pentyl butanoate, propyl hexanoate, ethyl 3-hydroxybutanoate, butyl propanoate) and 1-acetoxy-propan-2-one are crucial aroma components contributing to the characteristic fruity, fresh and sweet notes of durian [4,36]. Alpha-terpinene is known for its citrus and lemon scent [37], while benzyl alcohol exudes a honey-like sweetness and floral aroma, and both possess reported antioxidant and preservative properties, respectively [38,39]. Previous research has demonstrated that essential oils containing alpha-terpinene exhibit antimicrobial activity against *Salmonella enterica* [40]. Additionally, alpha-terpinene has been found to enhance the competitive ability of an environmental bacterium (*Le*YC36) in bacterial-fungal interactions by stimulating the production of antifungal agents [41], shedding new light on microbial communication and cooperative adaptation induced by SO_2_ in durian. 2-decanone is a natural volatile compound produced by fungi and insects. Studies have shown that it exhibits potent antifungal activity, effectively inhibiting brown rot disease in peach fruits caused by *Monilinia fructicola* [7] and controlling the soilborne pathogen *Sclerotinia sclerotiorum* in Coptis chinensis rhizomes [42]. Furthermore, 2-heptylfuran possesses complex odor characteristics including green, fatty, and milky notes, and demonstrates antioxidant properties that can inhibit benzo[a]pyrene-induced tumor development [43]. In contrast, signals in the green region weakened under the TS treatment. Throughout the storage period, 2-phenylacetaldehyde, ethyl 2-hydroxy-4-methylpentanoate, and heptanol were reduced in TS-treated samples. Among these, 2-phenylacetaldehyde is suggested to influence the aroma of mead [44]; ethyl 2-hydroxy-4-methylpentanoate is a compound directly associated with a “fresh blackberry” aroma in red wines [45]; and heptanol is reported as a flavor profile in beef, derived from lipid oxidation [46], although these compounds are rarely reported in fruits and vegetables. Previous studies have indicated that the content of volatile sulfur compounds increases with durian ripening [47]. In this study, 2,4,5-trimethylthiazole and 2-methyl-4-propyl-1,3-oxathiane, both sulfur-containing heterocyclic compounds, showed lower signal intensities in TS-treated samples at 15 days, suggesting that SO_2_ retarded the ripening and senescence of durian, thereby enhancing the fruit’s disease resistance. Compared to the initial sample (0 d), the control group on day 15 exhibits a marked enrichment of 5-methyl-2-furanmethanol, tetrahydrolinalool, isomenthone, isobutyl butyrate, alpha-terpineol, (Z)-4-decenal, beta-linalool, beta-phenylethyl alcohol, (E, E)-2,4-hexadienal, 10-undecenal and 2-methyl-4-propyl-1,3-oxathiane. This observation suggests that prolonged storage time contributes to increased durian maturity, with these VOCs playing a crucial role in the fruit’s complex flavor profile. In contrast, the TS-treated samples maintain relatively stable levels of these compounds throughout the storage period. These observations suggest that SO_2_ treatment may influence durian flavor by changing the levels of specific esters and compounds with antioxidant and antibacterial properties, but these effects still need to be further investigated. This modulation not only aids in preserving the distinctive aroma of durian but also improves the fruit’s resistance and prolongs ripening and senescence.

Differential spectra (Figure 3C) were derived by subtracting the In_0d spectrum, with red and blue areas denoting higher and lower VOC concentrations compared to In_0d, respectively. In comparison to the control group, samples treated with SO_2_ exhibited red-to-yellow areas at retention times of 300–500 s before day 10, which diminished by day 15, indicating changes in VOC composition following SO_2_ treatment, consistent with the findings from the fingerprint plot analysis (Figure 3B). Overall, over time, the T1 group demonstrated more consistent intensity and distribution of flavor compound signal peak, maintaining favorable signal characteristics throughout the storage duration.

### 3.3. Effect of SO_2_ (TS) Treatment on Fungal of Durian Husk

#### 3.3.1. TS Treatment Caused Shifts in Fungal Abundance of Durian Husk

Postharvest fungal infections cause significant losses in stored fruits and vegetables. Previous research has shown that SO_2_ treatment possesses antimicrobial properties when applied to fruits and vegetables [3]. This study focuses on ‘Montong’ durian and presents, for the first time, an analysis of the fungal community succession in durian husk influenced by SO_2_ treatment. Specifically, HTS of the internal transcribed spacer (ITS) was utilized to assess the impact of SO_2_ on the fungal community associated with durian husk. A total of 961 operational taxonomic units (OTUs) were identified through clustering at 97% sequence similarity. Analysis of the fungal rarefaction curve, rank abundance, and species accumulation boxplot showed that the sequence number was relatively abundant for observed species (Supplementary Materials, Figure S1).

**Figure 4:**
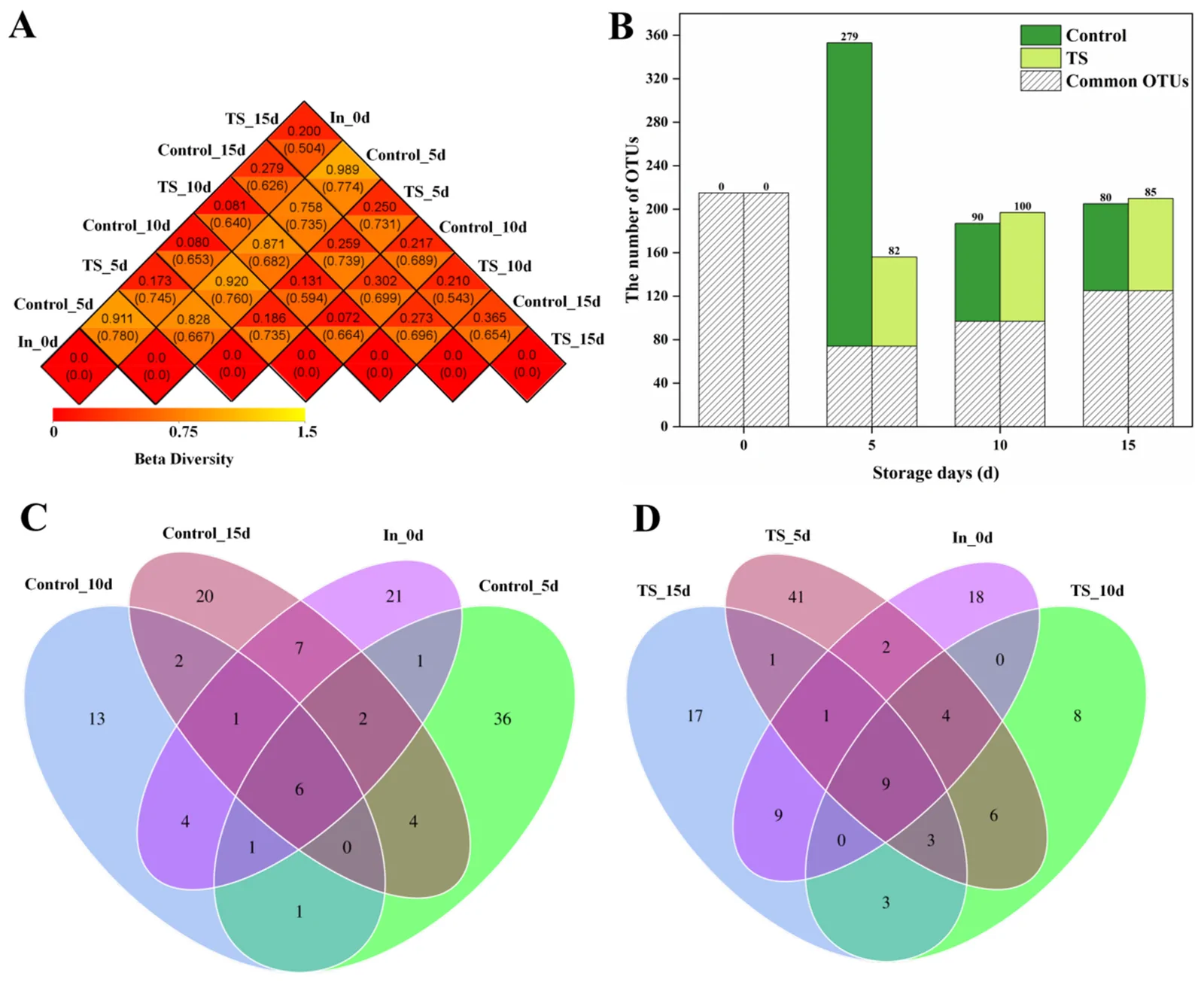
The heatmap of β diversity index, based on the distances of weighted UniFrac and unweighted UniFrac (**A**). The common and specific fungal OTUs on the surface of durian (**B**). The Venn diagrams based on OTUs at different storage times shown in (**C**) and (**D**), respectively.

The β-diversity index patterns differed between the TS treatment and the control, as shown by the UniFrac distance matrix presented in Figure 4A. The coefficients of weighted UniFrac and unweighted UniFrac were employed to assess species diversity between the two groups. A significant difference in β-diversity was observed between the TS treatment and the control (Figure 4). Excluding day 0, the weighted UniFrac distances between the two groups on days 5, 10, and 15 were 0.828, 0.072 and 0.365, respectively, while the unweighted UniFrac distances recorded were 0.667, 0.664, and 0.654. Notably, the weighted UniFrac distance reached its peak at day 5 before declining at day 10, whereas the unweighted UniFrac distances remained relatively stable. This pattern suggests that the SO_2_ treatment primarily influenced the relative abundances of shared taxa rather than inducing a complete turnover of species. The common and unique OTUs for the control and TS groups are illustrated in Figure 4B. In the initial sample (0 d), a total of 215 OTUs were identified. The numbers of unique OTUs in the TS group at 5, 10, and 15 days of storage were 82, 100, and 85, respectively. In contrast, the control group exhibited unique OTU numbers of 279, 90, and 80 at the corresponding time points. The unique OTUs in the TS-treated group exhibited fluctuations within a relatively narrow range throughout the storage period, peaking on day 10. In contrast, the control group displayed a marked increase in unique OTUs on day 5, reaching a total of 279, followed by a rapid decline on days 10 and 15. This pattern indicates that SO_2_ treatment mitigated the significant fluctuations in fungal community composition that occurred early in the storage of untreated fruit. As illustrated in Figure 4C,D, the number of OTUs shared across all storage time points, referred to as the core microbiota, was 9 in the TS-treated group, surpassing the 6 shared OTUs observed in the control group. This finding suggests enhanced community stability under SO_2_ treatment.

#### 3.3.2. TS Treatment Changed the Species Distribution of Durian Microbiota

**Figure 5:**
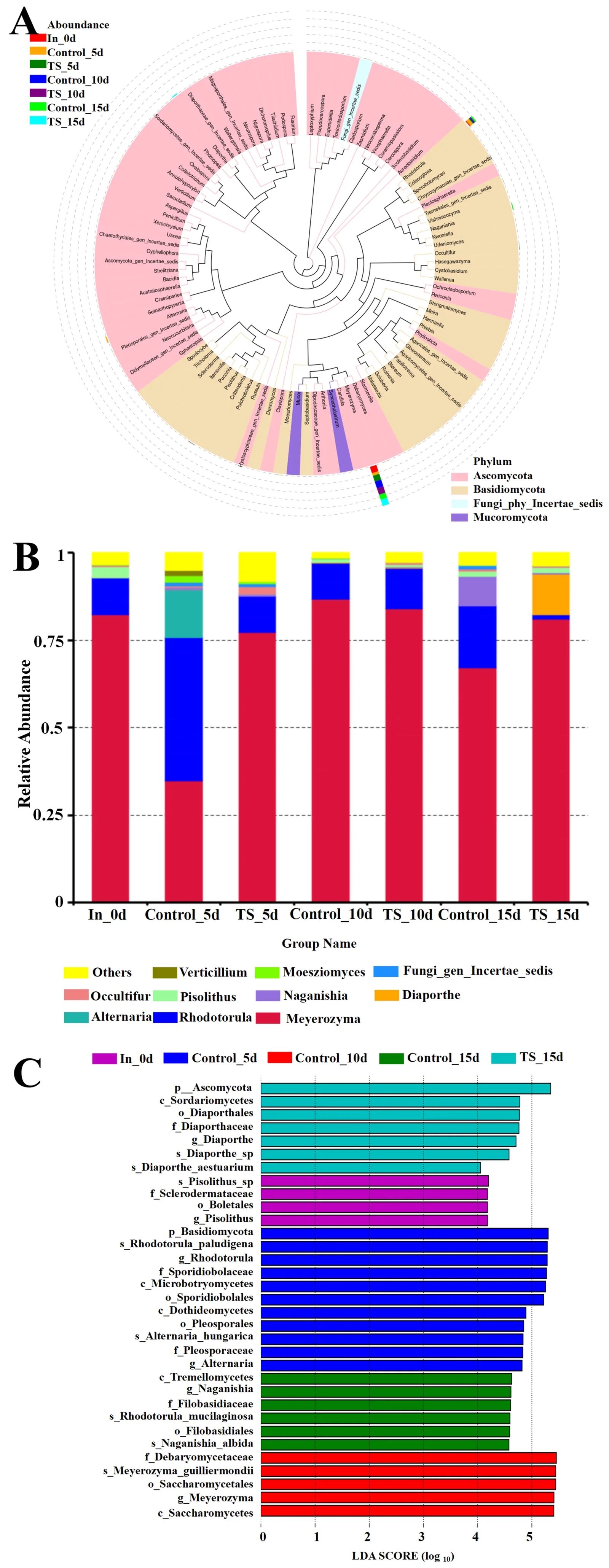
Phylogenetic trees for the top 100 genera across all samples (**A**). The relative abundance of the common taxa was shown at the genus level (top 10) (**B**). The potential fungal biomarkers as defined by LEfSe (**C**); p, phylum; c, class; o, order; f, family; g, genus; s, species.

The dynamics of microbial composition and relative abundance were represented through classification trees of the top 100 genera in Figure 5A, which revealed distinct fungal community structures within each of the two groups at varying storage durations. Notably, *Ascomycota* and *Basidiomycota* emerged as the predominant phyla identified on stored durian, with their relative abundance percentages accounting for 61.0–95.5% and 4.1–46.2% of the identified taxa, respectively.

At the genus level, the top 10 microbial taxa and their relative abundances are illustrated in Figure 5B and Table S2. Throughout the storage period, *Meyerozyma* sp. and *Rhodotorula* sp. emerged as the predominant genera in the control fruit. The relative abundance of *Meyerozyma* sp. was recorded at 82.42%, 34.76%, 86.87%, and 67.12% on days 0, 5, 10, and 15, respectively. For *Rhodotorula* sp., the relative abundances were 10.38%, 40.98%, 10.33%, and 17.87% on the same days. In TS-treated fruit, the relative abundance of *Meyerozyma* sp. was 82.42%, 77.22%, 84.08%, and 80.99% on days 0, 5, 10, and 15, respectively, surpassing that of the control fruit, except on day 10. *Meyerozyma guilliermondii* LMA-Cp01 is an ascomycetous yeast, with some strains utilized for the biocontrol of postharvest diseases in fruits and vegetables. This is achieved through the production of volatile antifungal metabolites, including alcohols, aldehydes, hydrocarbons, and terpene, which reduce disease incidence caused by pathogens such as gray mold (*Botrytis cinerea*) in apples, anthracnose (*Colletotrichum gloeosporioides*) in mangoes, and blue mold (*Penicillium expansum*) in peaches [48]. Additionally, it enhances the activities of defense-related enzymes, including POD, PAL, PPO, and β-GLU, thereby preventing and mitigating rot loss in harvested fruits [49]. The relative abundance of *Rhodotorula* sp. was recorded at 10.39%, 10.45%, 11.64%, and 1.31% on days 0, 5, 10, and 15, respectively, demonstrating relative stability until day 10. A decline in the relative abundance of *Rhodotorula* sp. was observed on day 15, accompanied by an increase in the relative abundance of *Diaporthe* sp. *Diaporthe* sp. is a known pathogen linked to postharvest decay in various fruits [50]; this finding may suggest that the efficacy of SO_2_ treatment in enhancing fruit disease resistance substantially outweighs the potential risks of decay associated with pathogen infection in durian. Previous studies have indicated that *Rhodotorula* sp. can induce reddish-brown discoloration on fruit surfaces [51], while also acting as a biocontrol agent that suppresses postharvest pathogens, thereby prolonging the storage life of fruits [52]. However, the specific mechanism of action of *Rhodotorula* sp. in durian fruit requires further investigation.

Differences also exist between the two groups for other species, including *Alternaria* sp., *Verticillium* sp., *Moesziomyces* sp., and *Occultifu* sp. On day 5, the relative abundances of *Alternaria* sp. (13.76%), *Moesziomyces* sp. (1.94%), and *Verticillium* sp. (1.48%) in control groups were higher than those in TS-treated samples. Conversely, *Occultifur* sp. demonstrated enrichment in TS-treated fruit. Notably, *Alternaria* sp. and *Verticillium* sp. were absent from TS-treated samples. *Alternaria* sp. is a primary pathogenic fungus responsible for postharvest rot in fruits and vegetables. Our findings align with previous research indicating that SO_2_ treatment effectively mitigates *Alternaria-*induced decay in grapes [53]. *Verticillium* sp., a highly destructive soilborne pathogenic fungus, primarily causes verticillium wilt, which results in significant yield loss and quality deterioration in fruits and vegetables [54]. Additionally, studies have identified *Moesziomyces* sp. as the principal pathogen responsible for fruit stain disease in grapes and pears [55]. *Occultifur* sp., a novel *cystobasidiomycetous* yeast [56], has been observed in the leaf spots of eggplant [57], and sugarcane leaves [56]. However, its functional roles remain unclear, and there is currently no direct evidence linking *Occultifur* sp. to postharvest diseases or the preservation of fruits and vegetables. Collectively, these findings indicate that SO_2_ treatment alters the fungal succession pattern in stored durian, significantly decreasing the relative abundance of spoilage and stain disease associated fungi, such as *Alternaria* sp., *Verticillium* sp., and *Moesziomyces* sp., while stabilizing beneficial yeast, especially *Meyerozyma* sp. This treatment does not eliminate all the molds, yeasts, and foodborne pathogens present on the durian’s surface; rather, it enhances the fruit’s defense against pathogens and preserves its quality.

Additionally, to identify the significantly different species in abundance (i.e., biomarkers) between the T1 and control groups, LEfSe analysis was conducted on microorganisms from the samples (Figure 5C). LEfSe revealed a total of 33 fungal taxa with significant differences. At the genus level, *Alternaria* sp. (5 d), *Rhodotorula* sp. (5 d), *Meyerozyma* sp. (10 d), and *Naganishia* sp. (15 d) were enriched in the control fruits. In contrast, *Diaporthe* sp. (15 d) was enriched at the genus level in TS-treated fruit. These results align with the analysis of species distribution.

### 3.4. Effect of SO_2_ (Ts) Treatment on CAT, PAL, POD, PPO, CHI and β-GLU Activities

**Figure 6:**
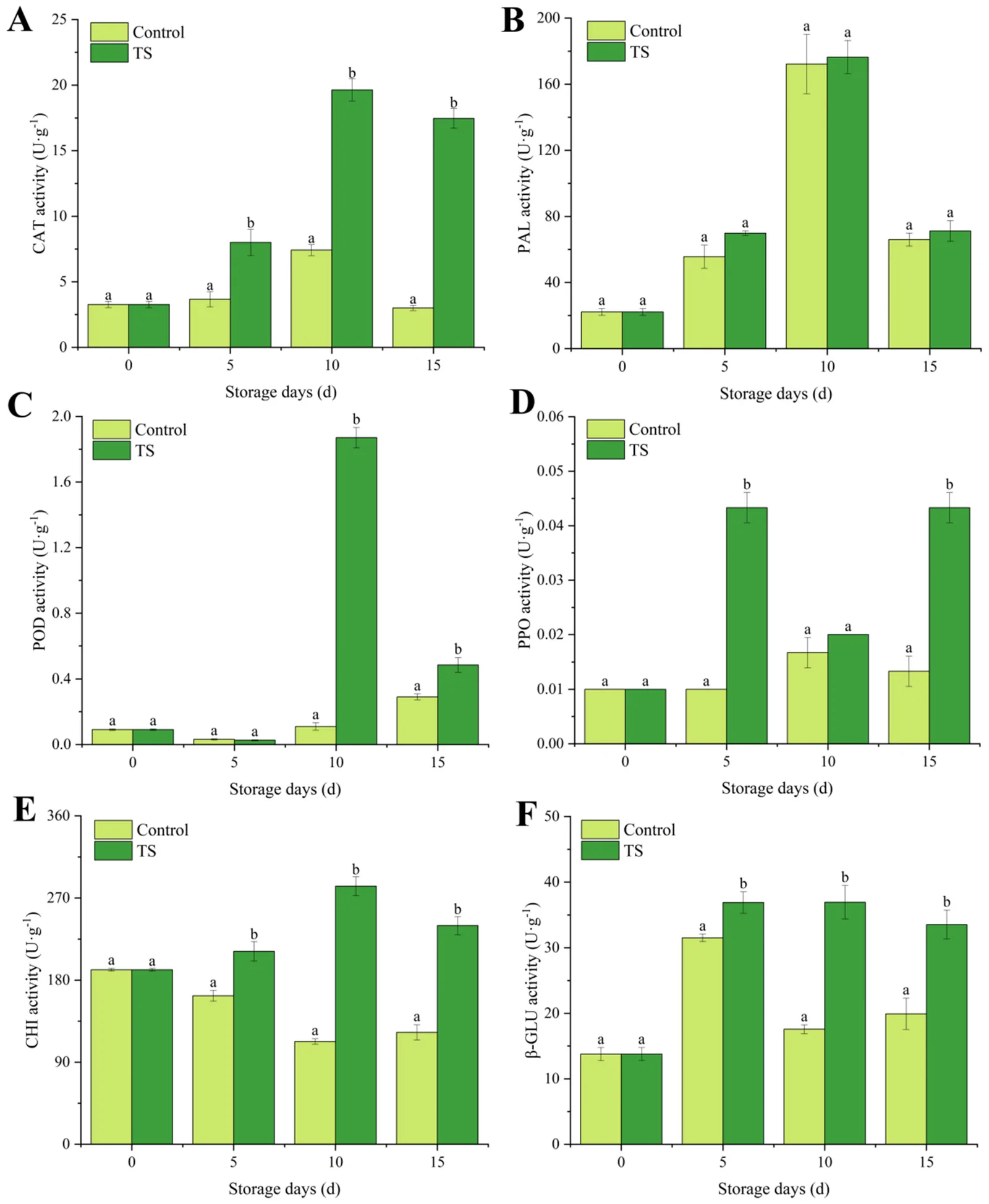
Effect of different treatments on CAT activity (**A**), PAL activity (**B**), POD activity (**C**), PPO activity (**D**), CHI activity (**E**) and β-GLU activity (**F**). Data are means ± SD of three biological replicates. Within the same storage day, different letters indicate significant differences between the control and TS samples (*p* ˂ 0.05; all pairs compared using an independent-samples *t*-test (two-tailed)).

The activation of defense-related enzymes in fruits and vegetables is considered a crucial factor in determining disease resistance [58]. In Figure 6A,C–F), during storage, the CAT, POD (except on day 5, not significant), PPO (except on day 10, not significant), CHI, and β-GLU activities in durian pulp of the TS group were significantly higher compared to those in the control group during storage (*p* < 0.05). However, the PAL activity changes in both TS-treated and control fruits showed no significant impact (Figure 6B) (*p* ˃ 0.05). Specifically, the patterns of defense-related enzyme activities varied in the control fruits. CAT, PPO and β-GLU activities initially increased and then decreased, while POD activity exhibited a gradual increase, and CHI activity demonstrated a steady decline. In contrast to the control group, PPO activity in the TS-treated group showed an initial increase on day 5, followed by a decrease on day 10, and a subsequent increase on day 15. Previous studies have highlighted the significance of PPO enzyme in animal and plant defense against bacterial and fungal pathogens, such as pea aphids [59] and cucumber fruit [60]. Therefore, on day 15, the PPO activity in TS-treated fruits increased sharply, which we hypothesize is related to microbial infection (e.g., *Diaporthe* sp., Figure 5B,C) that possibly triggered a secondary activation of the enzyme. Additionally, the CAT, POD, CHI, and β-GLU activities increased first and were followed by a decrease over the storage period, reaching peak levels on the 10th day in TS-treated fruits, which were 2.65, 17.00, 2.52, and 2.10 times higher than those in the control group, respectively.

Defense-related enzymes are crucial for managing postharvest pathogens, and their activities can be enhanced through the application of exogenous elicitors. For example, SO_2_ treatment activates the activities of PAL, PPO, CHI and β-*GLU,* while also elevating the transcription levels of pathogenesis-related protein genes *CHI3*, *CHIb*, and *β-GLU* (*PR2*) in grapes, thereby improving disease resistance [15]. Furthermore, SO_2_ treatment enhances the postharvest resistance of grapes to *Botrytis cinerea* by promoting antioxidant capacity [61]. This effect has also been observed in tobacco, where SO_2_ treatment increased superoxide dismutase (SOD) and POD activities, leading to a reduction in black shank disease caused by *Phytophthora nicotianae* [62]. Additionally, L-Phenylalanine effectively induces *Yarrowia lipolytica* to enhance the activities of defense-related enzymes, including PPO, POD, CAT, and CHI, thereby inhibiting blue mold in pears [63]. The antagonistic yeast, *Pichia caribbica,* can also elevate CAT, SOD, and POD activities, significantly controlling fruit diseases, such as blue mold rot in apples caused by *Penicillium expansum* [64] and black spot in tomatoes caused by *Alternaria* sp. [65]. These findings support our study’s conclusion that SO_2_ may increase the potential disease resistance of durian by inducing defense-related enzyme activities and altering the husk’s fungal community, which contribute to reducing infection by pathogenic fungi and improving the fruit’s storage quality. However, the causal relationships among VOCs, fungal succession, and defense-related enzyme activities were not established in this study and require further investigation.

## 4. Conclusions

Durian remains metabolically active after harvest, undergoing complex biochemical transformations that lead to diseases, aging, and flavor deterioration, which significantly reduce its market value. This research demonstrates that 2% SO_2_ suppresses respiration rates during the later storage period and reduces ethylene production, resulting in improved color, texture, flavor, and nutritional content of durian pulp. Furthermore, SO_2_ alters the husk’s fungal community (especially stabilizing the relative abundance of *Meyerozyma* sp.), enriches fruity esters (pentyl butanoate, propyl hexanoate, ethyl 3-hydroxybutanoate and butyl propanoate), antioxidant and antifungal VOCs, and activates the fruit’s defense-related enzyme activities. This study is the first to elucidate the potential coordinated responses among volatile metabolites, microbial community succession and enzymatic defense responses in SO_2_-treated fruit, thereby providing a scientific basis for its application in the postharvest preservation of durian.

## Figures and Tables

**Table 1:** Effect of different treatments on physicochemical properties of durian fruit during 14 °C storage.

Storage Days	Samples	Weight Loss Rate (%)	Firmness (N·cm^−2^)	L*	a*	b*	TSS (%)	Ascorbic Acid (mg·100 g^−1^)
0	Initial	0.00 ± 0.00 ^a^	0.20 ± 0.04 ^a^	85.10 ± 3.41 ^a^	−5.60 ± 0.19 ^a^	34.74 ± 1.98 ^a^	30.40 ± 0.00 ^a^	15.09 ± 0.41 ^a^
5	Control	3.89 ± 0.17 ^a^	0.08 ± 0.01 ^a^	85.01 ± 1.43 ^a^	−4.90 ± 0.15 ^a^	28.38 ± 1.22 ^a^	31.33 ± 0.23 ^a^	7.13 ± 0.51 ^a^
	TS	2.81 ± 0.34 ^b^	0.10 ± 0.01 ^b^	84.01 ± 0.82 ^a^	−5.44 ± 0.20 ^b^	30.32 ± 2.15 ^b^	27.60 ± 0.00 ^b^	9.36 ± 0.60 ^b^
10	Control	7.54 ± 0.10 ^a^	0.06 ± 0.01 ^a^	78.57 ± 2.79 ^a^	−4.52 ± 0.08 ^a^	29.60 ± 0.46 ^a^	29.47 ± 0.23 ^a^	6.96 ± 0.31 ^a^
	TS	6.13 ± 0.27 ^b^	0.09 ± 0.00 ^b^	83.64 ± 0.88 ^b^	−4.89 ± 0.22 ^a^	30.67 ± 0.66 ^b^	24.00 ± 0.00 ^b^	8.41 ± 0.73 ^b^
15	Control	15.83 ± 0.35 ^a^	0.07 ± 0.01 ^a^	76.81 ± 0.21 ^a^	−3.19 ± 0.14 ^a^	28.95 ± 1.37 ^a^	23.20 ± 0.00 ^a^	3.59 ± 0.59 ^a^
	TS	11.86 ± 1.16 ^b^	0.08 ± 0.00 ^a^	84.10 ± 0.66 ^b^	−3.40 ± 0.05 ^a^	35.32 ± 2.20 ^b^	30.13 ± 0.23 ^b^	6.71 ± 0.43 ^b^

Data are means ± SD of three biological replicates. Within the same storage day, different letters indicate significant differences between the control and TS samples (*p* ˂ 0.05; all pairs compared using an independent-samples *t*-test (two-tailed)). Initial: control sample, untreated with TS (0 d).

## Data Availability

Data is contained within the article or Supplementary Materials.

## Supplementary Materials

The following supporting information can be downloaded at: https://www.mdpi.com/article/10.3390/foods15193574/s1, Figure S1: The fungal rarefaction curve (A), rank abundance (B), and species accumulation boxplot (C); Table S1: Volatile organic compounds identified in durian pulp by GC-IMS at 14 °C storage; Table S2: The relative abundance of the dominant genera (top 10-group) of durian husk at 14 °C storage.
