# Supplementary material for: Effects of Sulfur Dioxide on Disease Resistance and Storage Quality of Durian (Durio zibethinus L.): A Comprehensive Analysis Integrating GC-IMS, HTS, Physiological and Biochemical Parameters
Source: Foods. 2026 Oct 8;15(19):3574. doi: 10.3390/foods15193574 (PMC13665371; doi:10.3390/foods15193574)
Supplement: Supplementary file 1 [file foods-15-03574-s001.zip › Table S2.pdf]

Table S2 The relative abundance of the dominant genera (top 10-group) of durian husk at 14 °C storage

Taxonomy	*Meyerozyma*	*Rhodotorula*	*Alternaria*	*Diaporthe*	*Naganishia*	*Pisolithus*	*Occultifur*	*Fungi_gen_Incertae_sedis*	*Moesziomyces*	*Verticillium*	*Others*
In_0d	82.42±4.30^{a}	10.39±2.21^{c}	0.00±0.00^{b}	0.00±0.00^{b}	0.17±0.00^{b}	3.20±2.66^{a}	0.29±0.10^{b}	0.00±0.00^{a}	0.17±0.17^{c}	0.00±0.00^{b}	3.37±1.99^{bc}
Control_5d	34.76±1.95^{c}	40.98±2.45^{a}	13.76±3.93^{a}	0.06±0.10^{b}	0.51±0.17^{b}	0.00±0.00^{b}	0.57±0.20^{ab}	0.91±0.52^{a}	1.94±0.20^{a}	1.48±0.55^{a}	5.02±0.71^{b}
TS_5d	77.23±13.00^{ab}	10.45±7.58^{c}	0.00±0.00^{b}	0.00±0.00^{b}	0.57±0.85^{b}	0.11±0.20^{b}	2.05±2.23^{a}	0.91±1.05^{a}	0.51±0.30^{b}	0.00±0.00^{b}	8.16±3.12^{a}
Control_10d	86.87±2.07^{a}	10.33±3.21^{c}	0.00±0.00^{b}	0.11±0.10^{b}	0.00±0.00^{b}	1.03±0.75^{b}	0.06±0.10^{b}	0.06±0.10^{a}	0.06±0.10^{c}	0.00±0.00^{b}	1.48±0.26^{c}
TS_10d	84.08±4.60^{a}	11.64±3.27^{bc}	0.00±0.00^{b}	0.17±0.00^{b}	0.29±0.10^{b}	0.51±0.30^{b}	0.63±0.26^{ab}	0.00±0.00^{a}	0.11±0.10^{c}	0.00±0.00^{b}	2.57±0.91^{bc}
Control_15d	67.12±5.41^{b}	17.87±3.63^{b}	0.00±0.00^{b}	0.00±0.00^{b}	8.45±5.06^{a}	1.37±0.34^{ab}	0.74±0.10^{ab}	0.86±0.79^{a}	0.11±0.10^{c}	0.00±0.00^{b}	3.48±1.17^{bc}
TS_15d	80.99±3.14^{a}	1.31±0.10^{d}	0.00±0.00^{b}	11.59±3.11^{a}	0.46±0.10^{b}	1.60±0.36^{ab}	0.34±0.30^{b}	0.06±0.10^{a}	0.00±0.00^{c}	0.00±0.00^{b}	3.65±0.43^{bc}

Data are means ± SD of three analytical replicates. Different letters indicate significant differences among samples ($p < 0.05$; one-way ANOVA).
