# Supplementary material for: Effects of Sulfur Dioxide on Disease Resistance and Storage Quality of Durian (Durio zibethinus L.): A Comprehensive Analysis Integrating GC-IMS, HTS, Physiological and Biochemical Parameters
Source: Foods. 2026 Oct 8;15(19):3574. doi: 10.3390/foods15193574 (PMC13665371; doi:10.3390/foods15193574)
Supplement: Supplementary file 1 [file foods-15-03574-s001.zip › Table S1.pdf]

Table S1 Volatile organic compounds identified in durian pulp by GC-IMS at 14 ℃ storage.

No.	Aroma composition name	CAS	RT(sec)	DT(a.u.)	Classes
1	Benzyl acetate	140-11-4	479,405	176,207	esters
2	Propyl hexanoate	626-77-7	425,358	139,231	esters
3	Pentyl butanoate	540-18-1	366,096	14,145	esters
4	Ethyl 4-oxopentanoate	539-88-8	330,015	164,572	esters
5	Ethyl 3-hydroxybutanoate	5405-41-4	219,871	116,555	esters
6	Isobutyl butyrate	539-90-2	221,252	133,166	esters
7	Ethyl butanoate	105-54-4	134,204	119,862	esters
8	Methyl butanoate	623-42-7	107,068	115,464	esters
9	Methyl 2-nonynoate	111-80-8	827,49	145,255	esters
10	Methyl anisate	121-98-2	914,779	181,369	esters
11	Ethyl 2-hydroxy-4-methylpentanoate	10348-47-7	367,747	130,571	esters
12	Butyl propanoate	590-01-2	180,404	128,912	esters
13	2,4,5-Trimethylthiazole	13623-11-5	275,806	156,197	sulfur compounds
14	2-Methyl-4-propyl-1,3-oxathiane	67715-80-4	470,88	171,036	sulfur compounds
15	Beta-linalool	78-70-6	395,727	168,586	alcohols
16	2-Furanmethanol, 5-methyl-	3857-25-8	233,682	155,778	alcohols
17	2-Heptanol	543-49-7	181,654	138,647	alcohols
18	Linalool oxide	1365-19-1	439,874	167,329	alcohols
19	Alpha-terpineol	98-55-5	595,594	178,835	alcohols
20	Beta-phenylethyl alcohol	60-12-8	432,992	128,611	alcohols
21	Heptanol	53535-33-4	217,547	1,396	alcohols
22	Benzyl alcohol	100-51-6	308,929	116,239	alcohols
23	Tetrahydrolinalool	78-69-3	396,492	127,557	alcohols
24	5-Methyl furfural	620-02-0	244,731	147,124	aldehydes

25	(E,E)-2,4-hexadienal	142-83-6	200,535	144,891	aldehydes
26	2-Furancarboxaldehyde	98-01-1	154,308	13,333	aldehydes
27	Hexanal	66-25-1	136,854	126,446	aldehydes
28	2-Phenylacetaldehyde	122-78-1	342,333	125,337	aldehydes
29	(Z)-4-Decenal	21662-09-9	596,432	134,486	aldehydes
30	Benzaldehyde	100-52-7	230,396	14,665	aldehydes
31	10-Undecenal	112-45-8	770,35	140,692	aldehydes
32	Octanoic acid	124-07-2	619,63	143,985	acids
33	Butanoic acid	107-92-6	126,322	116,247	acids
34	Isomenthone	491-07-6	535,117	134,504	ketones
35	2-Decanone	693-54-9	528,946	147,119	ketones
36	6-Methyl-3,5-heptadiene-2-one	1604-28-0	427,009	171,347	ketones
37	4-Ketoisophorone	1125-21-9	533,861	182,436	ketones
38	4-Methyl acetophenone	122-00-9	599,716	168,408	ketones
39	2-Undecanone	112-12-9	772,013	152,105	ketones
40	Alpha-terpinene	99-86-5	298,474	121,165	hydrocarbons
41	(-)-Beta-pinene	127-91-3	262,791	120,953	hydrocarbons
42	2,3-Dimethyl-5-ethylpyrazine	15707-34-3	364,327	173,706	heterocyclic compounds
43	2-Ethyl-3-methylpyrazine	15707-23-0	319,658	159,541	heterocyclic compounds
44	3-Ethylpyridine	536-78-7	251,982	152,707	heterocyclic compounds
45	3-Methyl-2-isobutyl pyrazine	13925-06-9	483,925	131,504	heterocyclic compounds
46	2-Heptylfuran	3777-71-7	620,115	139,418	heterocyclic compounds
47	2,5-Dimethyl-4-hydroxy 3(2H)- furanone (Furaneol)	3658-77-3	394,384	156,991	heterocyclic compounds
48	4-Methoxy-2,5-dimethyl-3(2H)-furanone	4077-47-8	316,468	154,705	heterocyclic compounds
49	4-Vinyl guaiacol	7786-61-0	828,411	12,239	others
50	1-Acetoxy-propan-2-one	592-20-1	174,691	119,672	others

51	Hexanenitrile	628-73-9	166,326	126,858	others

Note: RT, retention time; DT, drift time; CAS, Chemical Abstracts Service registry number.
