# Supplementary material for: Effects of Sulfur Dioxide on Disease Resistance and Storage Quality of Durian (Durio zibethinus L.): A Comprehensive Analysis Integrating GC-IMS, HTS, Physiological and Biochemical Parameters
Source: Foods. 2026 Oct 8;15(19):3574. doi: 10.3390/foods15193574 (PMC13665371; doi:10.3390/foods15193574)
Supplement: Supplementary file 1 [file foods-15-03574-s001.zip › Figure S1.pdf]

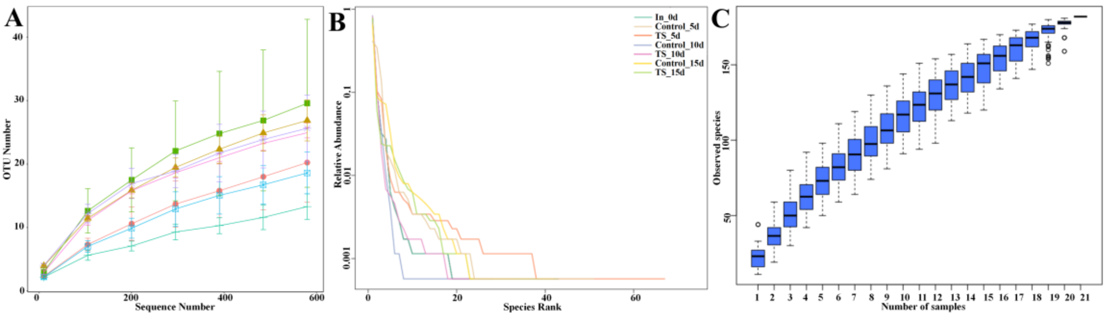

Figure S1. The fungal rarefaction curve (A), rank abundance (B), and species accumulation boxplot (C).
